# *IDA*-like gene expression in soybean and tomato leaf abscission and requirement for a diffusible stelar abscission signal

**DOI:** 10.1093/aobpla/pls035

**Published:** 2012-11-10

**Authors:** Mark L. Tucker, Ronghui Yang

**Affiliations:** Soybean Genomics and Improvement Lab, USDA/ARS, Bldg 006, BARC-West, 10300 Baltimore Avenue, Beltsville, MD 20705, USA

## Abstract

**Background and aims:**

The stimulatory and inhibitory role of ethylene and auxin, respectively, in leaf abscission (leaf drop) is well documented. More recently, IDA (INFLORESCENCE DEFICIENT IN ABSCISSION) peptides and their putative interacting receptor-like-kinase partners, HAESA and HAESA-like2, were shown to be essential components in *Arabidopsis* floral organ abscission. Prior to research on IDA, it was reported that bean (*Phaseolus vulgaris*) leaf abscission required a diffusible signal that emanated from the vascular tissue. We were interested in determining whether the IDA signalling path might regulate abscission in plants other than *Arabidopsis* and whether IDA might act as a diffusible signal in abscission.

**Methodology:**

Quantitative polymerase chain reaction was used to monitor gene expression and a GUS reporter gene construct used to determine the need for a diffusible signal in tomato.

**Principal results:**

We identified 12 *IDA*-like and 11 *HAESA*-like genes in soybean (*Glycine max*) and monitored their gene expression in abscission in relation to the expression of several cell-wall-modifying proteins and aminocyclopropane-1-carboxylic acid synthases. Ethylene evoked the expression of several *IDA*-like genes in abscission zones (AZ), but also to a lesser degree in the adjacent petiole tissue. Surprisingly, *IDA*-like gene expression was very high in senescent soybean leaves. We identified five *IDA*-like genes in tomato (*Solanum lycopersicum*). Only one *IDA*-like gene was expressed in the tomato AZ and its expression was approximately equal in the AZ and petioles, but no *IDA*-like gene showed significant expression in leaves at up to 96 h of exposure to ethylene.

**Conclusions:**

*IDA*-like gene expression is up-regulated during soybean and tomato abscission but up-regulation was not limited to the AZ. Cell separation in the AZ cortex of tomato does not require a diffusible signal emanating from the stele. A role for IDA in soybean and tomato leaf abscission is discussed.

## Abstract (precis)

IDA expression is essential for Arabidopsis abscission. Bean abscission requires a small signal that diffuses from the stele. We identified and examined the expression of multiple IDAs in soybean and tomato and their possible role as an abscission signal.

## Introduction

The stimulatory and inhibitory roles of ethylene and auxin, respectively, in controlling the onset of abscission (organ separation) have been known and studied for many years (Sexton and Roberts 1982; Sexton 1997; Roberts *et al.* 2002). More recently, additional regulatory components that are essential for floral organ abscission have been identified in *Arabidopsis* (Lewis *et al.* 2006; Nath *et al.* 2007). Butenko *et al.* (2003) identified an *Arabidopsis* abscission mutant and they named it *inflorescence deficient in abscission* (*ida*), in which the floral organs (petals, sepals and stamens) remain attached throughout enlargement of the silique. The *IDA* gene was found to encode a small protein that included an N-terminal signal peptide. In *Arabidopsis*, there are five more *IDA*-like (*IDL*) genes that are differentially expressed in development. When each *IDL* was overexpressed in *Arabidopsis*, all five *IDL*s displayed phenotypes similar to those of *IDA*-overexpressing plants, suggesting functional redundancy (Stenvik *et al.* 2008). The *IDA* and *IDL* genes all include a conserved (EPIP) peptide motif, and an exogenous application of synthetic EPIP peptide to *ida* mutant floral explants induced a nearly wild-type abscission response (Stenvik *et al.* 2008).

In an earlier study, Jinn *et al*. (2000) identified an *Arabidopsis* receptor-like kinase (RLK), which they called *HAESA*, that was highly expressed where the petals, sepals and stamens attached to the flower receptacle and, when suppressed in transgenic plants, abscission of the floral organs was delayed. Neither overexpression of the *IDA* gene nor exogenous application of the EPIP peptide rescued the delayed abscission phenotype in double mutants lacking functional *HAESA* (*HAE*) and *HAESA-like2* (*HSL2*) genes (Stenvik *et al.* 2008). Based on these data, Stenvik *et al.* (2008) proposed that the IDA peptide binds to HAE/HSL2 to initiate a signalling path that culminates in floral organ abscission. They also proposed that the other IDL peptides might act through RLKs like HAE/HSL2 to regulate events in other tissues and developmental processes. In the same year, Cho *et al.* (2008) corroborated the IDA–HAE/HSL2 interaction and extended the signalling path to include a mitogen-activated protein kinase cascade.

Here, we sought to determine whether *IDA* and *HAESA*-like gene expression in soybean (*Glycine max*) and *IDA*-like expression in tomato (*Solanum lycopersicum*) supported a possible role for these proteins in regulating abscission in these species. We analysed the expression of 12 *IDA*-like and 11 *HAESA*-like genes in soybean leaf abscission zones (AZ), petioles, leaves and roots, and five *IDA*-like genes in tomato leaf AZ, petioles, leaves, fruit and roots. To add perspective to the expression profiles for *IDA* and *HAESA*-like gene expression, we followed the expression of several genes for cell-wall-modifying proteins (CWMPs) previously demonstrated to be up-regulated and specific to abscission in soybean (Tucker *et al.* 2007) and tomato (Kalaitzis *et al.* 1997). In soybean, we also followed the expression of genes associated with the initial committed step for ethylene synthesis, aminocyclopropane-1-carboxylic acid (ACC) synthase (Tucker *et al.* 2010).

The interrelationship of *IDA* gene expression and ethylene is particularly important because although ethylene is not essential for *Arabidopsis* floral organ abscission (Patterson and Bleecker 2004), it appears to be essential in soybean and tomato (Lanahan *et al.* 1994; Roberts *et al.* 2002). With this in mind, we examined gene expression in AZ and petioles from explants kept in air without ethylene or exposed to air containing a physiologically high concentration of ethylene (25 μL L^−1^) or 2,5-norbornadiene (NBD), which inhibits ethylene action (Sisler 2006).

Thompson and Osborne (1994) proposed that bean leaf abscission requires a small signal produced in the vascular bundle (stele) that diffuses out into the cortex to initiate cell separation in the cortex. They observed that no endo-β-1,4-glucanhydrolase (cellulase) activity or cell separation was detectable in the AZ cortex if the cortex were separated from the stele prior to the treatment with ethylene; however, if they waited for several hours after treatment with ethylene and then separated the cortex from the stele, cellulase and cell separation in the cortex were detected. Because IDA is secreted and possibly processed into a smaller peptide (Stenvik *et al.* 2008), we hypothesized that the IDA peptide might be the small diffusible signal predicted to exist in bean leaf AZ (Thompson and Osborne 1994). Nevertheless, we first needed to determine whether a diffusible signal like that found in bean was essential for abscission in a species that we could easily surgically manipulate and which also had good molecular indicators for abscission. We chose to examine tomato leaf abscission to test for a requirement for a diffusible signal.

## Methods

### Tissue preparation

Stem/petiole explants ∼5–7 cm tall (leaf blades removed) were prepared from 2-week-old soybean (*G. max*, cv. Williams 82) seedlings grown in a growth chamber with 15 h of light at 23 °C, or from young (several weeks old) tomato plants (*S. lycopersicum*, cv. Ailsa Craig) grown in the greenhouse. Explants were placed in Erlenmeyer flasks of water inside a dark chamber maintained at 25 °C, where they were exposed to a gas flow of either air or 25 μL L^−1^ ethylene in air. For NBD treatments, the soybean explants in flasks were placed in 9-L desiccators and liquid NBD injected through a septum to achieve an NBD gas concentration of 2000 μL L^−1^. The desiccators were then placed in a dark chamber at 25 °C. The desiccators were opened every 48 h to collect tissue and when closed again the NBD was replenished. From the soybean explants, the AZ (∼2 mm) were harvested from the upper foliar AZ immediately below the leaf blade. In tomato, the lower AZ at the petiole stem juncture was collected. In soybean, the petiole material was excised from between the AZ at either end. In tomato, the petiole was collected ∼4 mm distal to the lower AZ. Leaves excised from the explants described above were placed on moist paper towels and exposed to 25 μL L^−1^ ethylene in air at 25 °C in the dark. Tomato fruit were collected from greenhouse plants at the ripening stages indicated. The entire root systems of greenhouse tomato plants were collected for RNA extraction. For soybean, sections of the root relative to the root apex (0–2, 2–7, 7–12 and 12–50 mm proximal to the apex) were collected separately as previously described (Tucker *et al.* 2007).

### Sequence identification and protein alignments

*IDA*-like and *HAESA*-like genes were identified in the genomic sequences for soybean and bean assembled by the Joint Genome Institute (JGI) and made available at **http://www.phytozome.net/soybean**. The *IDA* genes for tomato were identified in the genomic sequence deposited in the National Center for Biotechnology Information (NCBI). Sequence alignments were completed using MacVector ClustalW (MacVector Inc, Cary, NC, USA). Unrooted dendrograms were prepared using PAUP* Version 4.0 software (Sinauer Associates, Sunderland, MA, USA). Putative N-terminal signal peptides were identified using the SignalP 3.0 software available at **http://www.cbs.dtu.dk/services/SignalP/**.

### Quantitative polymerase chain reaction

Procedures for quantitative real-time polymerase chain reaction (QPCR) and the PCR primers used to examine gene expression for CWMPs were described previously (Tucker *et al.* 2007). A single bulk cDNA synthesis reaction (5 μg of DNased RNA) was performed and the cDNA diluted to 2.0 mL to accommodate a large number of PCR reactions and thereby reduce differences that might occur between cDNA synthesis reactions. Quantitative real-time polymerase chain reactions were completed using a Brilliant II SYBR Green QPCR Master Mix in an Mx3000P instrument (Stratagene, La Jolla, CA, USA). Soybean is a tetraploid and most genes are found as paralogous pairs with highsequence identity. Gene-specific primers for all the *ACS*, *IDA* and *HAE* genes were prepared by designing the primers to match less conserved parts of the sequences so that the 3′-end nucleotide of the primer was a mismatch between paralogous genes [Additional Information File AI2]. The 3′ mismatch generally prevents amplification of highly similar sequences. All QPCR Ct values were normalized to the expression for ubiquitin in soybean (accession AK285252) and tomato (accession BT012698). In the heat map display only, if the expression of a gene relative to ubiquitin was <0.0001, the expression value was set to 0.0001. A relative concentration of 0.0001 represents a PCR product detected at ∼35 cycles. Setting a lower limit of 0.0001 reduces potential artefacts associated with numerous PCR cycles and eliminates ratios with a denominator of zero.

## Results

### Gene identification and sequence comparisons

**Fig. 1:**
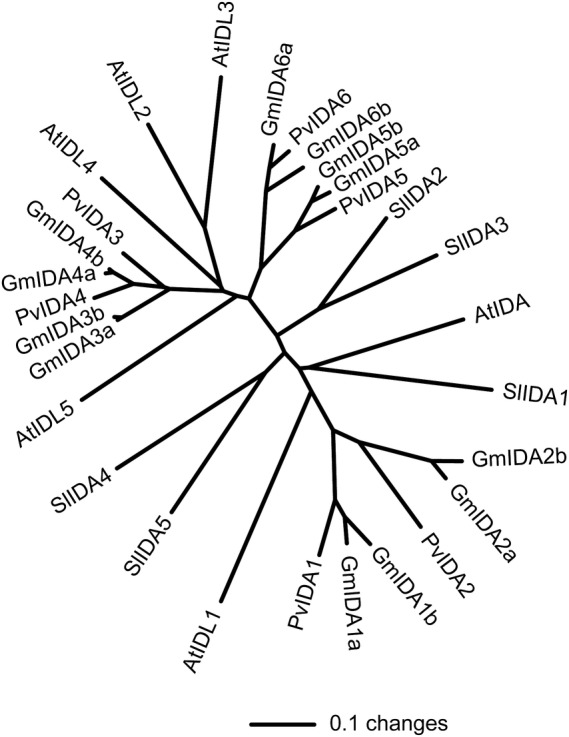
An unrooted dendrogram based on per cent amino acid identity among *Arabidopsis*, bean, soybean and tomato IDA and IDA-like protein sequences minus the N-terminal signal peptides.

**Fig. 2:**
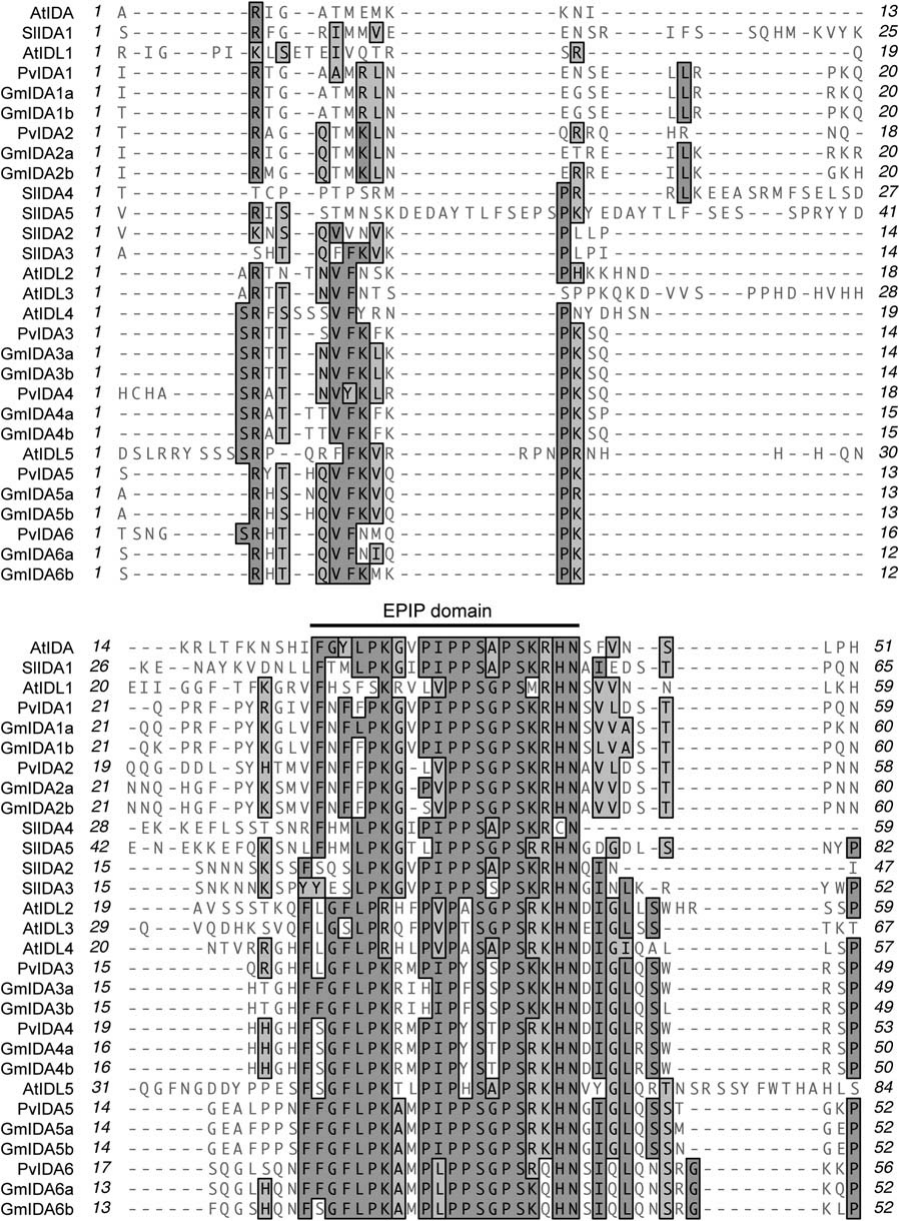
**Alignment of translated open reading frames minus a predicted N-terminal signal peptide for *Arabidopsis*, bean, soybean and tomato IDA and IDA-like sequences.** Identical conserved amino acids are enclosed in grey boxes and similar amino acids are enclosed in lighter grey boxes.

We identified in the soybean genomic sequence 12 *IDA*-like genes (henceforth referred to simply as *IDA*). The soybean *IDA* genes were identified using a TBLASTN search (six-frame translation) of the *G. max* genomic sequence with the *AtIDA* (accession NP564941) protein sequence minus its N-terminal signal peptide. The 12 soybean *IDA* sequences were named *GmIDA* 1 through 6 with the letter a or b appended to denote highly similar paralogous genes. A similar approach was used to identify six *IDA*-like genes in bean (*Phaseolus vulgaris*) and five in tomato. The relatedness of the six *Arabidopsis*, 12 soybean, six bean and five tomato IDA proteins is displayed in the dendrogram shown in Fig. 1 [nucleotide sequences available in Additional Information File AI1]. All the *IDA* genes include an uninterrupted open reading frame with no introns and encode a translation product with a predicted N-terminal signal peptide sequence. The amino acid sequence similarity between AtIDA and other IDA-like proteins minus the putative signal peptides ranges from 24 % with AtIDL3 to 53 % with GmIDA1a. All of the IDAs include a variable region immediately after the signal peptide, and all include the highly conserve EPIP domain (Stenvik *et al.* 2008) (Fig. 2).

**Fig. 3:**
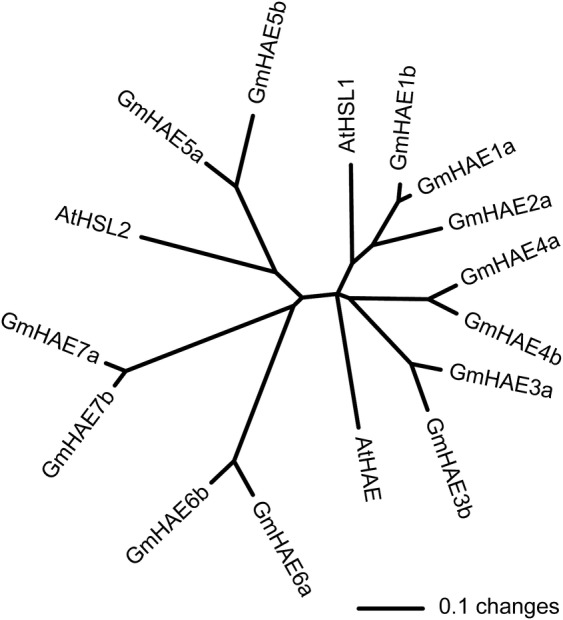
An unrooted dendrogram based on per cent amino acid identity among *Arabidopsis* and soybean HAE and HSL protein sequences.

Eukaryotic genomes commonly include many *RLK* genes (Shiu and Bleecker 2001; Morris and Walker 2003). Assuming that the translated open reading frames for the soybean genes most similar to the *Arabidopsis* HAESA (HAE) and HAESA-like2 (HSL2) peptides retained IDA-ligand-binding specificity and kinase signalling, we performed a TBLASTN search of the soybean genomic sequence with the AtHAESA and AtHAESA-like peptide sequences. Thirteen soybean *HAESA*-like genes (henceforth referred to simply as soybean *HAE*) were identified. The relatedness of the 13 soybean HAE and the *Arabidopsis* HAE and HSL proteins is displayed in the dendrogram shown in Fig. 3 [nucleotide sequences provided as Additional Information File AI1]. The soybean sequences identified and selected for study ranged from 82 % amino acid similarity between GmHAE1a and AtHSL1, and 49 % amino acid sequence similarity between GmHAE7a and AtHSL2. Within the protein sequences, the most highly conserved region was in the protein kinase domains in the C-terminal third of the protein.

### Reference genes

**Fig. 4:**
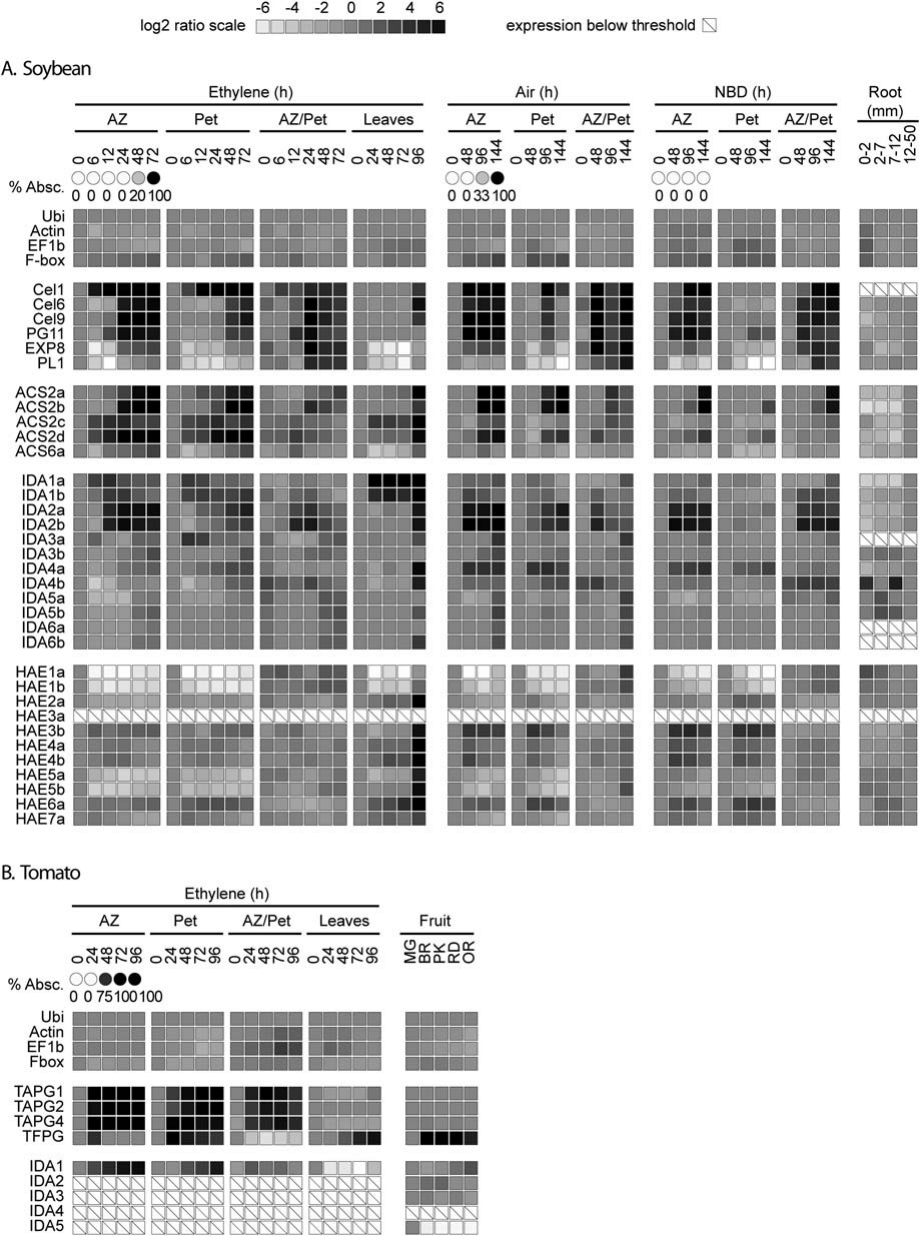
**Heat map displaying the change in gene expression (log base 2 ratios) for soybean (A) and tomato (B) genes in abscission zones (AZ), petioles (Pet), leaves, fruit (tomato only) and roots (soybean only).** The soybean root pieces were collected as 0–2, 2–7, 7–12 and 12–50 mm sections proximal to the root apex. AZ and Pet treatments were 25 μL L^−1^ ethylene in air, air, or 2000 μL L^−1^ 2,5-norbornadiene (NBD) in air. The QPCR results shown here were all normalized to soybean and tomato ubiquitin (soybean AK285252, tomato BT012698). A dark box indicates strong up-regulation of gene expression whereas a white box indicates strong down-regulation, and no change in expression is indicated by a neutral grey box (see the scale at the top). The log2 ratios for tissues labelled as AZ or Pet are ratios for the expression at the indicated time over the zero-time collection (0 h). The log2 ratios labelled AZ/Pet are the ratios for the expression in the AZ relative to the expression in the petioles at the indicated time of collection. Gene name abbreviations for the CWMPs are: cellulase (Cel), expansin (EXP), pectate lyase (PL) and polygalacturonase (PG). The tomato fruit PG (PG2a) is indicated as TFPG and the abscission PGs as TAPG. An a, b, c or d after an ACS gene indicates that this group of genes are highly similar to the same numbered gene in *Arabidopsis* (Tucker *et al.* 2010).

The expression profiles for all the genes analysed here were determined by QPCR. The choice of a reference gene is important to the interpretation of QPCR results (Czechowski *et al.* 2005; Lilly *et al.* 2011). In this regard, we examined the expression of four commonly used reference mRNAs for soybean and tomato: ubiquitin (AK285252, BT012698), actin (AK285258, AK322149), elongation factor 1 beta (AK243885, AK246849) and F-box (Glyma18g51130.1, AK327900) (Czechowski *et al.* 2005; Lilly *et al.* 2011). The ubiquitin Ct (threshold value) varied the least among the four; nonetheless, the expression of all the reference genes was relatively constant in the tissues examined (Fig. 4). Because ubiquitin expression varied the least of the four, all the QPCR results were normalized and made relative to the concentration of ubiquitin in the respective soybean and tomato RNA populations.

### Expression profiles for soybean IDAs and other genes

**Fig. 5:**
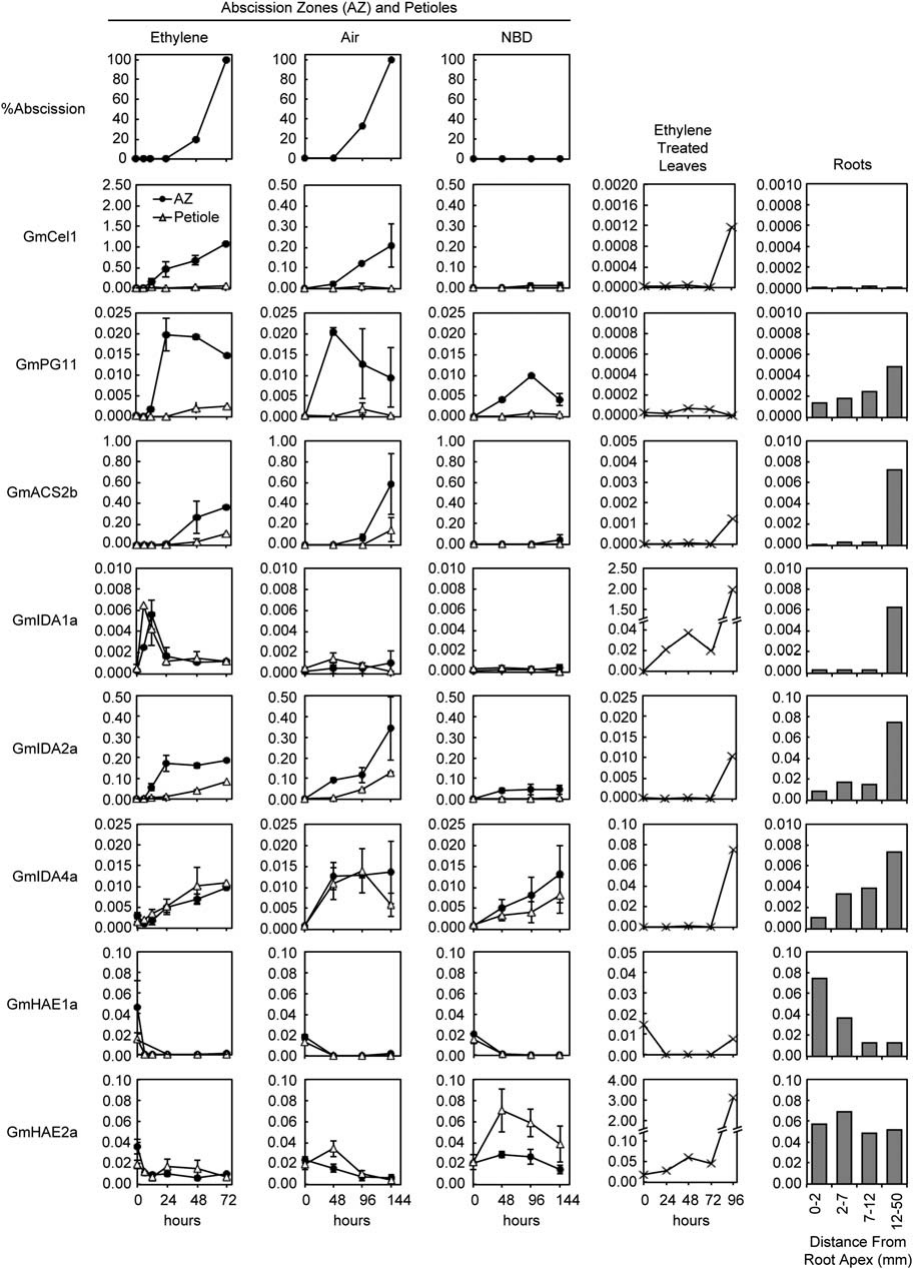
**Time-dependent graphs for per cent abscission in soybean explants and gene expression profiles for genes selected based on significant change in their expression either in abscission or roots.** Treatments and normalization are described in Fig. 4. Note that the scale changes for some graphs in order to better illustrate differences in gene expression between treatments. The means and standard error bars for AZ and petioles (Pet) are for two independent replicate experiments. All others are single experiments that included many AZ, petioles, leaves and root pieces.

**Fig. 6:**
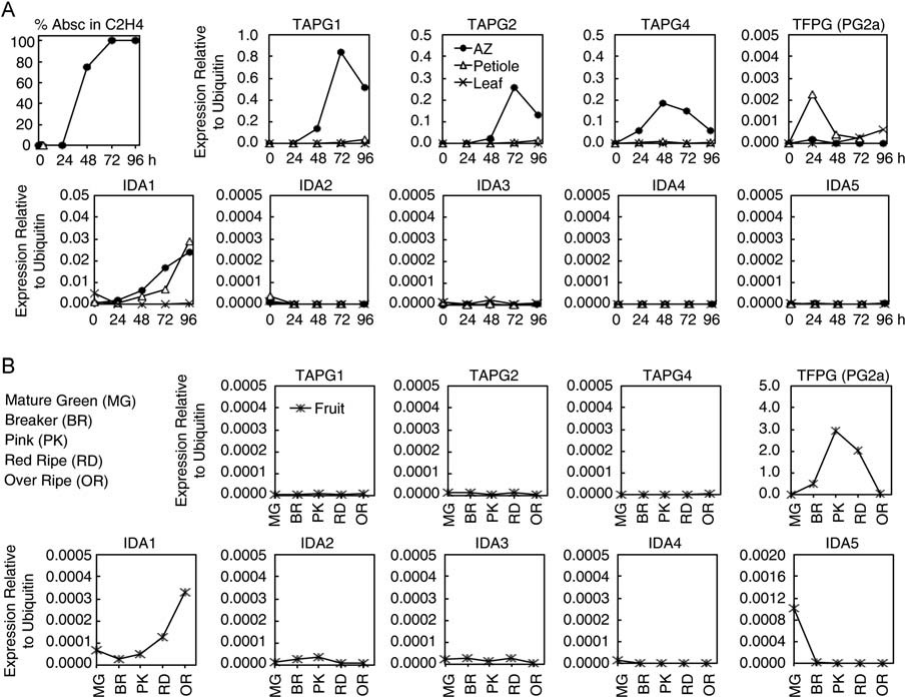
**Time-dependent graphs for per cent abscission in tomato explants and gene expression profiles for AZ, petioles (Pet), leaves and fruit.** As in Fig. 4, the QPCR results were all normalized to ubiquitin. The data points are for a single experiment that included many AZ, petioles, leaves and fruit. Fruit stages are: mature green (MG), breaker (BR), pink (PK), red ripe (RD) and over ripe (OR).

For soybean, we monitored the mRNA accumulation of 32 CWMPs (Tucker *et al.* 2007), 17 ACC synthases (Tucker *et al.* 2007), 12 IDA and 11 HAE proteins. However, for brevity, we only display here the results for six CWMPs and five genes for ACC synthases (*ACS*) that showed significant change in abscission (Fig. 4). To further understand how ethylene affects the expression of each of these soybean genes, we exposed the stem/petiole explants to air without ethylene, air + 25 μL L^−1^ ethylene or air + 2000 μL L^−1^ NBD, a competitive inhibitor of ethylene action (Sisler 2006). After 48 h exposure to ethylene, 20 % of the AZ had separated (abscised) and after 72 h 100 % had abscised (Fig. 5). In air, separation lagged behind the ethylene-treated explants by ∼48 h, showing only 40 % separation at 96 h and 100 % after 144 h (Fig. 5). An increase in gene expression for most of the selected cell wall proteins can be seen within 12 h after exposure to ethylene or at 48 h when explants were kept in air (Figs 5 and 6). 2,5-Norbornadiene completely blocked abscission for 144 h but only decreased gene expression for the same cell wall proteins by ∼90 % as compared with similar explants kept in air (Fig. 6).

ACC synthase is essential for ethylene synthesis (Yamagami *et al.* 2003). The expression of several *ACS* genes increased markedly in the ethylene-treated AZ at approximately the same time as the increase in *Cel1* and *PG11* (Figs 4 and 5). The increase in expression of *ACS2* isoforms (a, b, c and d) was particularly marked. However, the increase in *ACS* gene expression was not as AZ specific (expressed in the AZ relative to the petiole) as were the CWMPs (Figs 4 and 5). In air, the increase in expression of *ACS* seemed to lag behind the CWMPs. An increase in *ACS* gene expression was not noted in air until 96 h, and NBD suppressed expression of *ACS* even more than it did the CWMPs. However, it is worth noting that *ACS* gene expression was easily detected at our 0 h time point in AZ, petioles and leaves. At our 0 h time point, the expression relative to ubiquitin of *ACS2d*, *ACS6a*, *ACS6b*, *ACS9a*, *ACS9b* and *ACS9d* was > 0.001.

The expression of *IDA2a*, *IDA2b* and *IDA4a* increases markedly during abscission in the soybean explants, and *IDA2a* and *IDA2b* were more highly expressed in the AZ than in petioles (Figs 4 and 5). Expression of all the soybean *IDA*s tended to lag behind the early expression of *Cel1* and was more similar to the expression pattern for the other cellulases and *PG11* (Fig. 4). Interestingly, up-regulation of some of the *IDA* genes was less affected by the NBD treatment (Fig. 5). *PG11* expression was also less affected by the NBD treatment (Fig. 6).

Several soybean *IDA*s were also expressed in roots (Figs 5 and 6). Interestingly, the *IDA*s up-regulated in the AZ were not highly expressed near the growing root tips but further back in the root, which suggests they might be associated with lateral root initiation. However, other *IDA* genes, i.e. *IDA3b*, *IDA5a* and *IDA5b*, were most highly expressed immediately behind the root meristem (Fig. 4) where cell elongation and vascular differentiation occur (Tucker *et al.* 2007). Also of interest is the observation that *IDA* expression increased markedly in senescent soybean leaves after 96 h of ethylene (Figs 4 and 5). *IDA1a* and *IDA1b* displayed especially high expression late during senescence, even more abundant than ubiquitin (Fig. 5). Interestingly, although five *IDA* genes were identified in tomato, only *IDA1* increased significantly in tomato abscission explants and the increase was equal in both the AZ and petiole (Fig. 6). Although the tomato *IDA1* transcript was detected in leaves (Fig. 5B), it did not display the very large increase in expression observed for soybean after 96 h exposure to ethylene. IDA gene expression was also detected in the root system of tomato (results not shown) but a detailed expression relative to the root apex was not performed.

In *Arabidopsis*, HAE and HSL2 putatively act as redundant receptors for the IDA peptide in a signalling path that induces gene expression leading to separation of the floral organs (Cho *et al.* 2008; Stenvik *et al.* 2008). In soybean, we identified the genes most similar to the *Arabidopsis HAE* and *HSL2* genes (Fig. 3) and examined their expression (Figs 4 and 5). Based on the expression patterns for the soybean *HAE* genes, there is no clear indication that any of these proteins play a special role in abscission (Fig. 4). Soybean *HAE2a* expression was included in Fig. 5 because its expression, like that of *IDA1a* and *IDA1b*, is very strong in senescent leaves. It is possible that the IDA1a and/or IDA1b peptides interact with HAE2a to regulate some part of senescence in leaves.

### Cell-to-cell signalling

**Fig. 7:**
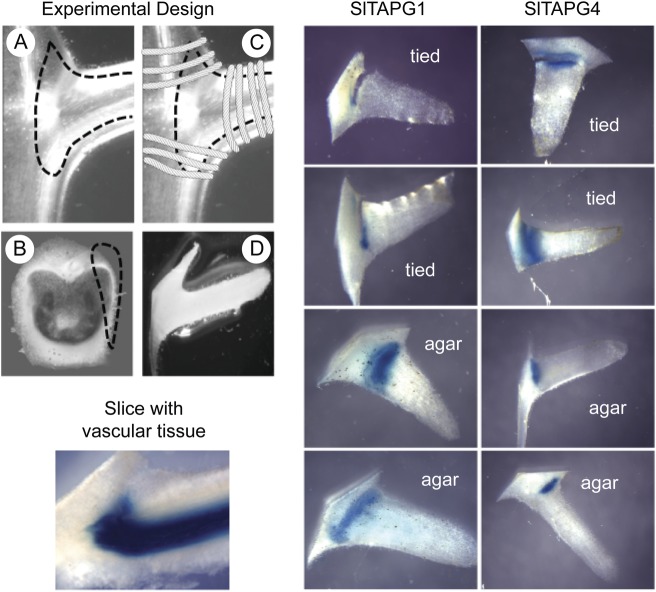
**Protocol and images for GUS-stained tomato leaf AZ.** The TAPG1::GUS and TAPG4::GUS transgenic plants used for these experiments were described previously (Hong *et al.* 2000). Side slices of AZ cortex were prepared from intact stem/petiole explants (A). A depiction of how the side slice was prepared to avoid vascular tissue is shown in the GUS-stained cross-section of an abscised AZ (B). The slice was either tied back onto the same position on the explant (C) or placed on agar (D). Explants with slices tied back on and slices on agar were exposed to 25 μL L^−1^ ethylene in air at 25 °C for 90 h. After 90 h, side slices were collected from explants (tied) and agar (agar), and stained for GUS activity. An example of a side slice kept on agar that included some vascular tissue is shown to demonstrate how slices that included vascular tissue could be identified and eliminated from analysis.

Results with bean leaf abscission indicated that a small molecular signal was produced in the vascular tissue of bean AZ that diffused out from the stele to induce cell separation in the cortex (Thompson and Osborne 1994). We hypothesized that this signal might be the IDA peptide, but we first needed to determine whether cell-to-cell signalling was required in a system for which we had good markers and that we could surgically manipulate. We chose to use the tomato polygalacturonase 1 (*TAPG1*) and 4 (*TAPG4*) promoters ligated to a GUS reporter gene as indicators for cell separation in tomato (Hong *et al.* 2000). Before treatment with ethylene, we sliced off a piece of the cortex at the AZ of a tomato stem/petiole explant and then either tied the slice back onto its original position on the explant or placed the slice on agar (Fig. 7). After 90 h of ethylene exposure, the side slices were collected from the explants and agar, and stained for GUS expression. If by mistake the cortex slice included some of the vascular bundle, this was easily detected after ethylene treatment because both *TAPG1* and *TAPG4* expression in the vascular tissue extends up the vascular bundle a few millimetres distal to the separation layer (Hong *et al.* 2000). Vascular expression of TAPG4::GUS in a side slice can be seen in an example included in Fig. 7. In the case of tomato leaf abscission, cell separation and GUS expression occurred in both the slices that were tied back onto the AZ or placed on agar (Fig. 7).

## Discussion

In *Arabidopsis*, *IDA* gene expression signals the beginning of floral organ abscission and appears to be essential for abscission (Butenko *et al.* 2003). IDA::GUS expression in *Arabidopsis* is generally localized to the region where abscission will occur but not precisely restricted to where separation occurs (Butenko *et al.* 2003, 2006). *IDA*-like transcripts in species other than *Arabidopsis* were found in EST databases for several different plant species (Butenko *et al.* 2003) but their expression profiles have not been extensively studied. One objective of the experiments described here was to identify *IDA*-like genes in species other than *Arabidopsis* and examine their expression to determine whether IDA might perform a similar function in these species. In soybean, a tetraploid legume, we identified 12 *IDA*-like genes and in bean, a diploid legume, we identified six (Figs 1 and 2). We also identified five *IDA*-like genes in tomato (Figs 1 and 2).

Many years ago, Jackson and Osborne (1970) demonstrated that ethylene increased in the AZ of petioles, and a role for ethylene in abscission has been confirmed by many others (Taylor and Whitelaw 2001; Roberts *et al.* 2002). Aminocyclopropane-1-carboxylic acid synthase is essential for the biosynthesis of ethylene (Yamagami *et al.* 2003). Thus, we examined the gene expression profiles for 17 soybean *ACS*. Many genes for CWMPs have been demonstrated to increase during abscission and as a group are essential for cell separation (Roberts *et al.* 2002). A few CWMPs whose expression is highly specific to the AZ were included in the current study to add perspective to the profiles for *IDA*, *HAE* and *ACS* gene expression. Although *ACS* expression was somewhat higher in the AZ of soybean, it was not as AZ specific as the genes for the selected CWMPs (Fig. 4). Whether or not *ACS* is AZ specific, the rise in *ACS* gene expression in explants kept in air and NBD came after the rise in several genes for CWMPs and *IDA* (Fig. 4). Of interest in this regard is that gene expression for several *ACS* genes was low but easily detected by QPCR in AZ at the beginning of each treatment (0 h). This suggests that although ethylene biosynthesis increases during abscission, a change in the concentration of other abscission-inducing signals must precede the increase in ethylene that sensitizes the AZ to low levels of pre-abscission ethylene or activate ethylene synthesis from pre-existing ACC synthases. As noted previously, a decrease in auxin is essential for abscission to occur and the decrease in auxin might be the signal for an increase in ethylene synthesis and other early changes in gene expression (Tucker *et al.* 1988; Taylor and Whitelaw 2001; Roberts *et al.* 2002), but are there additional signals, e.g. IDA, that play a primary role in leaf abscission of soybean and tomato?

We monitored the expression of 12 soybean *IDA*s and for most of the *IDA* genes expression increased late in abscission. However, by far the greatest and earliest increase occurred for *GmIDA2a* and *GmIDA2b*, each increasing by more than 100-fold in the AZ after 24 h of ethylene treatment or 96 h in air. The increase in *GmIDA2a* and *GmIDA2b* transcript was relatively specific to the AZ but somewhat less AZ specific than the genes for the CWMPs (Figs 4 and 5). However, if you add up expression levels for all the *IDA* genes and assume that the secreted peptides are functionally redundant, there is a fairly high level of total *IDA* transcript in both AZ and petioles that could lead to synthesis and secretion of an active IDA peptide. However, the amount of IDA peptides secreted into the apoplast may not be directly proportional to the amount of *IDA* transcript in the AZ or petiole, and the receptor for these ligands may not be distributed in the same pattern as the secretion of the IDA peptide. Of interest in this regard is the finding that overexpression of *IDA* in *Arabidopsis* activated vestigial abscission of leaves (Stenvik *et al.* 2006). This suggests that a receptor for IDA is present in the vestigial leaf AZ that is capable of inducing an AZ-specific cell separation response. Thus, it may be the receptor that determines AZ-specific signalling.

In *Arabidopsis*, IDA putatively interacts with the HAE and HSL2 RLKs (Cho *et al.* 2008; Stenvik *et al.* 2008). Expression of *AtHAE* and *AtHSL2* promoter::GUS constructs in *Arabidopsis* indicated that these genes are expressed in the floral organ AZ but not the surrounding tissue (Jinn *et al.* 2000; Cho *et al.* 2008). GUS expression from the *AtHAE* promoter was first apparent in floral AZ when flowers were competent for pollination and its expression was similar in the ethylene-insensitive mutant *etr1-1*, which indicates that its expression was independent of ethylene (Jinn *et al.* 2000). Thus, for soybean, we also examined the expression of potential receptors for the IDA peptide, i.e. *HAE*-like genes. None of the soybean *HAE*-like genes identified had a transcript expression pattern that was specific to the AZ (Fig. 4), and therefore, even if they might be receptors for an IDA peptide, these RLKs probably do not define a separation layer within the AZ. Other RLKs or other molecules or proteins must be responsible for defining those cells within the AZ that can respond to abscission signals.

In addition to soybean, we also quantified the expression of five tomato *IDA*-like genes (Fig. 6). Only tomato *SlIDA1* increased significantly in leaf AZ and the increase was mirrored in the petioles. *SlIDA1* also increased slightly during the ripening of tomato fruit but the abundance of the transcript was considerably less than in the AZ. None of the tomato *IDAs* increased in the ethylene-treated leaves, which was different from soybean. However, after 96 h of ethylene exposure the tomato leaves were not as yellow as the soybean leaves at the same time point; it is possible that *IDA* might have increased in tomato leaves if they were exposed to ethylene for a longer time.

In *Arabidopsis*, IDA is secreted into the apoplast, where it may be further processed into an even smaller peptide (Stenvik *et al.* 2008). It is possible that IDA could diffuse short distances in the apoplast of the AZ or be actively translocated across AZ cells. When onion-skin cells were bombarded with an IDA–GFP fusion construct, GFP fluorescence was observed in several neighbouring cells, which indicated diffusion through the apoplast or non-specific translocation across neighbouring cells (Butenko *et al.* 2003). Thompson and Osborne (1994) proposed that the stele in the AZ of bean produced a diffusible molecule that was necessary for initiation of cell separation in the cortex. McManus (2008) extended this earlier result to demonstrate that the diffusible stelar substance by itself was not sufficient to induce separation in the cortex but that ethylene was also essential. We hypothesized that the relatively small IDA peptide might be the diffusible molecule predicted by Thompson and Osborne (1994). However, before testing this hypothesis, we needed to find a model system that we could use. We already had transgenic tomato seed that included a GUS reporter gene ligated to the *TAPG1* or *TAPG4* promoters (Hong *et al.* 2000). We also had a transgenic soybean which includes a PG11::GUS construct which is expressed in soybean AZ (Tucker *et al.* 2011). The tomato AZ are quite large and it was fairly easy to slice off the side of the AZ to separate the cortex from the stele; however, this was not so easy in the much smaller soybean AZ. When we sliced off the tomato AZ cortex and placed it on agar, the cortex expressed GUS and displayed cell separation when exposed to ethylene much the same as when the cortex slice was tied back onto its original position on the side of the AZ (Fig. 7). The result for tomato side slices indicates that there is no need for a diffusible signal from the stele. We recently found an older publication by Roy Sexton (1979) where he dissected the foliar AZ of *Impatiens sultani* into many smaller pieces and placed the pieces on agar. After 30 h at 22 °C he examined each piece, many of which did not include vascular tissue, for cell separation. He concluded that ‘there was little requirement for cell to cell contact in either the temporal or spatial integration of cell wall breakdown’ in the AZ of *I. sultani*. We conclude that a diffusible stelar signal similar to that discovered in bean is not universally required for leaf abscission; however, because of experimental limitations with soybean, we cannot conclude that a diffusible signal is not required for soybean cortex abscission. This remains to be tested in future experiments.

As a part of this project we examined the expression of *IDA* and *HAE* in ethylene-treated leaves. Unexpectedly, expression of several soybean *IDA*s increased late in senescing (yellowing) leaves exposed to ethylene for 96 h. Most notably, *GmIDA1a* and *GmIDA1b* transcripts accumulated to very high levels in senescent leaves (Figs 4 and 5). Interestingly, there was a corresponding large increase in the expression of *GmHAE2a* in senescent soybean leaves (Figs 4 and 5). What role IDA signalling might play in senescence is unknown and how much of the gene expression changes observed in the petiole and AZ of the soybean and tomato explants can be linked to a general senescence response is also unknown.

With regard to identifying a function for IDA signalling in plants other than *Arabidopsis*, the dendrogram for the IDAs might shed some light on this (Fig. 1). The dendrogram suggests that multiplication of the *IDA* and *IDA*-like genes may have occurred after divergence of *Arabidopsis* (Brassicaceae), tomato (Solanaceae) and soybean (Fabaceae), but before divergence of soybean and bean, both in the Fabaceae family. The fact that *Arabidopsis* includes six *IDA* and *IDL* genes, and we identified six genes in bean (a diploid), 12 in soybean (a tetraploid) and five in tomato (a diploid) may be coincidental. It is possible that the IDA signalling mechanism itself is what is important to the plant, and the signalling mechanism was duplicated and adapted for use in diverse developmental processes in different plant families.

## Conclusions and forward look

Although ethylene appears to be essential for abscission in many species (Tucker *et al.* 1988; Lanahan *et al.* 1994; Roberts *et al.* 2002), it is not essential for floral organ abscission in *Arabidopsis* (Butenko *et al.* 2006). In *Arabidopsis*, conversely to ethylene, IDA and its putative binding partners HAE/HSL2 appear to be essential to floral organ abscission (Jinn *et al.* 2000; Butenko *et al.* 2003). *IDA* gene expression increases many fold during soybean and tomato leaf abscission, but based on gene expression patterns alone, we cannot conclude that IDA signalling is required for abscission in these species. In tomato, IDA gene expression was approximately equal in the AZ and petioles and, in soybean, IDA expression was slightly more AZ specific but not so obviously specific to the AZ to justify a conclusion that IDA signalling is necessary for abscission in soybean. In experiments where we put the soybean explants in water with 10 μM EPIP peptide and exposed the explants in the peptide solution to air or ethylene, we did not observe any effect on the rate of abscission (results not shown). Suppression of IDA gene expression in transgenic plants will be necessary to determine whether or not IDA signalling plays a primary role in soybean or tomato abscission.

Shi *et al.* (2011) proposed that IDA signalling in *Arabidopsis* abscission affects cell enlargement through its regulation of a subset of KNOX transcription factors. In this regard, Meir *et al.* (2010) showed a strong decrease in two *KNOX* genes in tomato pedicel AZ when the flowers were removed, which also occurred in AZ when ethylene action was inhibited with 1-MCP. The decrease in *KNOX* gene expression might be caused by a decrease in auxin but might also be linked to IDA signalling. A possible link between IDA and regulation of KNOX transcription factors in soybean and tomato needs to be examined.

Assuming sequence conservation between *Arabidopsis* HAE and HSL2 proteins and RLKs with a similar role in soybean, we attempted to identify RLK partners for soybean IDAs. Based on gene expression patterns, it is possible that soybean IDA1a and IDA1b might interact with soybean HAE2a late in senescence, but none of the expression patterns for the 11 RLKs that we examined seemed to fit with our expectations for an IDA ligand receptor involved in soybean abscission. Sequencing of the soybean transcriptome may provide a more refined list of RLK candidates that might interact with IDA in an abscission response and warrant further examination.

## Additional information

The following additional information is available in the online version of this article –

**File AI1**. *IDA* and *HAESA* sequences.

**File AI2**. Primers used for QPCR experiments.

## Contributions by the authors

M.L.T. designed the experiments, conducted some experiments and prepared the manuscript. R.Y. did all the QPCR experiments and helped with the interpretation of the results.

## Conflict of interest statement

None declared.
